# Preparing for success in final summative medical specialist examinations: The case for RACE

**DOI:** 10.1186/s12909-023-04920-y

**Published:** 2023-12-05

**Authors:** Penelope Allen, Belinda Jessup, Melissa Kirschbaum, Santosh Khanal, Victoria Baker-Smith, Barnabas Graham, Tony Barnett

**Affiliations:** 1 Rural Clinical School, College of Health and Medicine, University of Tasmania, Burnie, TAS Australia; 2 Centre for Rural Health, College of Health and Medicine, University of Tasmania, Locked Bag 1322, Launceston, TAS 7250 Australia; 3 The Royal Australian and New Zealand College of Ophthalmologists, Sydney, NSW Australia

**Keywords:** Medical specialist examinations, Specialist trainees, Registrars, Ophthalmology, Specialist training College, High-stakes summative examinations

## Abstract

**Background:**

Failure rates on medical specialist final summative examinations in Australia are high, regardless of speciality. Examination failure can have detrimental psycho-social, financial and job security effects on the trainee, while delays in completion of training adversely impacts workforce growth and health outcomes for the community. The study aimed to explore the preparation factors that contribute to ophthalmology trainee success in their final summative examination.

**Methods:**

Semi-structured in-depth interviews were conducted with 29 participants via telephone or Zoom with ophthalmology trainees and Fellows. To be eligible, interviewees had to have sat the Royal Australian and New Zealand College of Ophthalmologists Advanced Clinical Examination (RACE) within the past five years or were providing supervision to trainees preparing for RACE. Interviews were audio-recorded, transcribed and thematically analysed.

**Results:**

Examination success was underpinned by six themes relating to preparation: (i) ‘Those who fail to plan, plan to fail’, which related to development and adherence to a study plan; (ii) ‘It takes a village’ encompassed trainees establishing and activating personal and professional supports; (iii) ‘Get to know your opponent’, which encompassed developing an understanding of the examination construct, format and requirements; (iv) ‘There is no substitute for hard work’, which related to intensive study over a period of 12–18 months; (v) ‘Keep pace with the herd’, which referred to benchmarking preparation efforts and progress against peers; and (vi) ‘Don’t jump the gun’, which related to ensuring readiness to sit.

**Conclusions:**

Maximising medical specialist examination pass rates is in the best interest of trainees, training Colleges, health care systems and communities. Recognising and facilitating preparation approaches that foster success in final summative examinations are the collective responsibility of trainees, specialist training Colleges, training networks and health systems. Trainees need to plan for examination success, be self-determined to commit to intensive study over an extended time period and be realistic about their readiness to sit.

**Supplementary Information:**

The online version contains supplementary material available at 10.1186/s12909-023-04920-y.

## Background

Assessment during postgraduate medical training is essential to ensure that specialists entering the workforce are competent and ready for independent practice. For many medical specialties, there is an emphasis on high-stakes summative examinations, which commonly take place towards the end of the training program [1]. In the case of ophthalmology training in Australia and New Zealand, the Royal Australian and New Zealand College of Ophthalmologists (RANZCO) Advanced Clinical Examination (RACE) is a summative hurdle assessment comprised of a written and clinical component, which determines progression to final year of training [2]. In recent years there has been concern about RACE failure rates, in particular the written component [3, 4]. Analysis of recent RACE results as part of a larger study found the percentage of candidates who passed the written exam ranged from a low of 30% in semester 2, 2017 to a high of 81% in semester 1, 2018, while the pass rate for the clinical component ranged from 56% in semester 2, 2018 to 90% in semester 2, 2020 [4].

Postgraduate medical examinations are known to be particularly stressful events [3–6]. The experience of failure can have detrimental mental health, financial and job security effects on the trainee [5, 7]. In addition, resultant delays in completion of training adversely impact the specialist medical workforce. Minimising the failure rate is therefore not only in the best interests of trainees, but also for specialist medical Colleges and the community who stand to benefit from an increased medical specialist workforce. Concomitantly, it is imperative that clinical and professional standards are maintained and trainees who pass summative examinations meet curriculum requirements [2, 8].

Failure rates on final summative examinations are high, regardless of specialist medical College [1, 4, 9–13]. This has prompted research which has identified a range of potential reasons for failure, including trainee personal factors and examination preparation factors (such as study strategies and juggling clinical workloads) [1, 4, 9, 13–20]. Despite this research, there is a relative lack of published literature around preparation strategies that promote success in postgraduate summative medical specialist examinations. Some studies suggest strategies such as involvement in a study group, greater total study hours, studying a range of materials, participation in courses, using past examination questions for practise, and proficiency in examination technique are important for success [4, 14–17, 19, 21]. The aim of our study was to contribute further to the understanding of preparation approaches that support examination success by exploring the factors that contributed to ophthalmology trainees passing RACE. The findings are anticipated to provide trainees across all specialist medical Colleges with guidance on key approaches to preparation that promote success on final summative examination.

## Methods

### Setting

RACE is the final summative examination of the Australian and New Zealand ophthalmology vocational training program, overseen by RANZCO. Basic and advanced training is centred on work-based learning with trainees employed as accredited registrars. Trainees are rotated through various clinical settings and subspecialties, across seven training networks. Trainees must have satisfactorily completed three years of vocational training and have passed previous summative and formative assessments to be eligible to sit RACE [2, 4]. The examination comprises a written (online) component consisting of two papers, each with Short Essay Questions and Very Short Answer Questions. The questions are weighted for equal representation from the nine clinical curriculum performance standards. The clinical component consists of an Objective Structured Clinical Examination (OSCE), with individual stations assessing clinical application of curriculum knowledge. Both components of RACE must be successfully completed before progression to the final year of training, thus allowing trainees to graduate as ophthalmologists once their final year of training is complete. There are two opportunities to sit the examination each year and trainees may attempt the examination up to three times.

### Study design and ethical approvals

As part of a larger study, semi-structured in-depth interviews were conducted with ophthalmology trainees and Fellows across Australia and New Zealand. The University of Tasmania Human Research Ethics Committee (Project ID: 25018) and RANZCO Human Research Ethics Committee (Reference: 129.21) provided ethical approvals for the research.

### Recruitment and data collection

Eligible interviewees were ophthalmology trainees or Fellows who had sat RACE within the past five years (≥ 2017) (n = 166) and RANZCO Fellows supervising trainees preparing for RACE (n = 127). Potential participants were recruited via email invitation sent by a member of the research team (BJ).

A semi-structured interview guide, developed by the research team, was used to elicit information on preparation for the final summative exam and participants’ experiences leading up to, and during the examination (Supplementary file). Three sources of information were accessed in the development of the interview guide to ensure validity of the questions. (1) The research team read the RANZCO RACE protocols and policy documents. (2) Quantitative and qualitative data from several years of past examination feedback surveys (collected from RACE candidates immediately after the written and clinical examinations) were analysed to identify specific areas of concern about the examination from the candidates’ perspectives. (3) The research team consulted with an expert panel, consisting of members of the Board of Examiners and the RANZCO education team. The expert panel provided information on the structure and content of RACE, and shared their perspectives on examination difficulty and pass rates over time. Minutes of the meeting were recorded and used as a data source for construction of the survey questions. Additionally, expert panel participants were requested to email the research team with questions that they considered were important to include in the interview guide. The draft list of interview questions was reviewed by the research team and circulated to the expert panel for feedback prior to being finalised.

Interviews were conducted via Zoom or telephone by one researcher (BJ) during January-March 2022. All participants provided verbal consent to participate and for their interview to be audio-recorded. Interviews ranged from 27 to 75 min. Interview recordings were transcribed verbatim and transcripts were emailed to participants to check for accuracy and clarify any component of the transcript. Once confirmed, transcripts were anonymised by assigning a label (e.g. trainee or supervisor) and number (e.g. 1). Any identifying information inadvertently stated by interviewees during the recording was removed from the transcripts prior to analysis.

### Data analysis

Transcripts were imported into NVivo version 12.0 for analysis [22]. The interview data were analysed according to the methodology espoused by Braun and Clarke [23]. Firstly, individual transcripts were reviewed multiple times by members of the research team (BJ, PA, TB). Using an inductive approach, BJ and PA independently read the transcripts and identified codes as they emerged from the text by selecting the relevant text in the transcript (saved as data files in NVivo) and assigning a code to the selected text. Codes were given draft names as they were generated, and a description of the code was entered. The codes were hierarchically organised into sub-themes, and eventually into overarching themes as the coding progressed. Both researchers then met to discuss their coding and to confirm the main categories, sub-categories and the relationships between categories. While there were no instances of overt disagreement in coding or categories, there were differences in the naming and describing of themes. These were resolved through consensus discussion between the university researchers (BJ, PA, TB), with each researcher having equal weight in the discussion. To avoid a potential conflict of interest, the study team members employed by RANZCO were excluded from data collection and analysis.

## Results

Twenty-nine ophthalmologists (12 women and 17 men) who had sat RACE recently (n = 19) or were supervisors of trainees preparing to sit RACE (n = 10) were interviewed. Participants were representative of all seven RANZCO training networks across Australia and New Zealand. Eight trainees shared experiences of failure on one or more sittings of RACE, and all supervisors had supervised trainees who failed RACE.

Analysis of the interview data identified six key themes regarding trainee preparation that promoted examination success (Table 1).

**Table 1:** Key themes related to trainee preparation that promoted examination success and recommendations for action

Theme	Theme meaning	Recommendations for training Colleges
***‘Those who fail to plan, plan to fail’***	Developing a structured study plan and sticking to it.	Develop and provide trainees with an examination orientation pack at least 12 months before being eligible to sit the examinations. Provide trainees with a recommended study plan.
***‘It takes a village’***	Establishing and activating personal and professional supports.	Assign a study adviser/study coach (professional mentor) to each trainee. Review and disseminate guidelines to all Fellows and consultants on how to better support trainees preparing to sit examinations.
***‘Get to know your opponent’***	Understanding the examination construct, format and requirements.	Implement a virtual whole of College information and preparation course to all trainees at least 12 months before being eligible to sit. Establish virtual whole of College teaching sessions (e.g. tutorials, mock OSCEs). Provide trainees with recent examination papers, together with model answers, examiner feedback and marking rubrics.
***‘There is no substitute for hard work’***	A lengthy period of intense study is essential.	Advocate for workplaces to support trainees in scheduling a period of annual leave prior to commencing examination preparation. Encourage trainees to implement psycho-social supports to facilitate intensive study.
***‘Keep pace with the herd’***	Benchmarking preparation efforts and progress against peers.	Ensure trainees have equity of access to a study group. Strengthen mechanisms for advanced trainees to be identified prior to commencing each rotation, so that supervisors can adequately prepare and support exam preparations. Advocate for workplaces to support trainees by allowing leave to attend examination preparatory courses.
***‘Don’t jump the gun’***	Ensuring readiness to sit before attempting the examination.	Ensure all trainees who submit ‘intention to sit’ paperwork have a face-to-face meeting with their relevant network Director of Training to assess ‘readiness to sit’.

### ‘Those who fail to plan, plan to fail’

Interviewees reported that they started to prepare for RACE years in advance. Most interviewees commented that a study plan or timeline (developed individually or within study groups) was the essential first element in their preparation. Study plans provided the structure, discipline and accountability that kept them focussed and motivated over the long study period up to the examination. Most trainees suggested that structuring the timetable around the curriculum standards proved useful to ensure the comprehensiveness of study.

*We had a conference and we sat down and said, ‘look, this will be the biggest examination we’re going to sit in our lives, we want to get through the first time so let’s come up with a strict study schedule and let’s not waste time anymore’. (Trainee 8)*

*In terms of preparing for the examination, I set a timetable very, very early on in the piece and that basically involved me starting 11 months prior to sitting the written examination… I think organization, planning, writing it all down at the very beginning, going through the curriculum, it helped us immensely, and we spent like six hours doing that … and then we just sort of stuck to the plan. (Trainee 3)*

Trainees who were in locations with heavy workloads/on-call demands when trying to prepare reflected on their frustrations in attempting to secure study time. Some trainees were wise to this issue, and as part of their planning requested training rotations in locations which would support their preparations through minimal on-call, a flexible roster that facilitated attendance at tutorials and teaching sessions, access to Fellows with examination committee experience, and approval of study leave to attend residential examination preparation courses.

*The only thing I found really tricky [when preparing for RACE as a trainee] was I was on-call for six months straight up, on-call 24/7. Like there was no weekend off. No nothing. Because some of the rotations, you alternate week on week off, but the ones I was on there was only one registrar there and so you’re on-call the whole time. So I found that really difficult. There wasn’t a point where I could just plan, ‘okay, Sunday I’ll do all day’, because you’d end up being called in and then frustrated with yourself because you like only had two hours today. (Supervisor 1)*

*I had a rotation at the sort of main tertiary hospital at a time when the juniors were pretty good and it was very well staffed and so I’d just finish my clinic and go study straightaway and come in a bit late in the morning and study in the morning. I had plenty of extra time to study. (Trainee 17)*

### It takes a village

The experiences shared by interviewees demonstrated that preparation for RACE was underpinned by collective efforts and support from the trainees’ entire training network, the College and their family and friends. First, trainees in their basic years of training were seen as important in supporting sitting candidates by taking up extra duties in clinics which increased study opportunities for trainees preparing for RACE. Interviewees acknowledged the importance of reciprocity of collegiality in the network.

*There’s a good culture in [network] amongst the registrars themselves, that like you scratch my back, I scratch yours. So I’m a junior registrar, I know you’re sitting your exams in a couple of months, so I’ll step up and do a lot more of the clinic and give you time to sort of go off to this tutorial, or go off to this teaching session, or take some time off from the clinic so you can prepare a bit more. And then when it’s my go, I know you’ll be through and you’ll hand me your notes or give me some time so you cover me when I need to do my examination. (Supervisor 9)*

Second, final year trainees who had already passed RACE contributed to trainee preparation by providing important advice for RACE preparation through their lived-experience of either examination success or failure. Trainees in their final year of training were also seen as a vehicle for study notes; although not all were willing to share their resources with sitting candidates. Trainees emphasised that the study plans used by previous cohorts of successful RACE candidates provided a starting point which current candidates could adapt to suit their own plans.

*We just asked the people in the year above, ‘How did you do it?’ And then we took their plans and went, well that doesn’t quite work for us, because we’re all away in different places, we’re going to do it this way instead. So we just changed it to suit our lifestyles, our personal situations, and of course, our work locations and situations a bit more. And that really helped. (Trainee 3)*

*I got resources from a number of people, probably had too many, more than what I needed. But then a number of other trainees have asked me for what I’ve had, so I’ve downloaded what I have in a big folder and given it to them to work their way through. Because it took me a long time to acquire it. And look, some of the registrars don’t really want to share their stuff with anyone else and other people will. (Trainee 14)*

Third, interviewees described that trainees needed to surround themselves with others preparing to sit the examination to build an emotional support network. This was often achieved through the formation of study groups which provided camaraderie, emotional and moral support and the opportunity for peer encouragement. Of note, smaller training networks meant some trainees were left isolated as there were no other trainees preparing to sit. A few of these trainees described overcoming this challenge by connecting virtually with other trainees in an online study group.

*I think having that peer support and the combined learning experience with colleagues going through exactly the same thing as you are at exactly the same time, that’s the single biggest thing that I think we need to make sure they all have. Because without that, I honestly don’t think you can pass. I really don’t. (Supervisor 5)*

*There’s so much importance in having a group of people who not only help you study and study together and share knowledge, but it’s just camaraderie, team building, personal support. (Trainee 6)*

*The trouble with being in [location] is that we are all scattered all over …so it’s difficult … now since the pandemic, a blessing in disguise, we’ve had Zoom. So we’re meeting up every week on Zoom. (Trainee 5)*

Fourth, Fellows and supervisors were pivotal given they helped trainees prepare by quizzing them on their knowledge during clinics and surgeries. Supervisors and Fellows also provided much needed feedback regarding the adequacy and accuracy of trainee answers when using previous exams for practise.

*When I was at [hospital] just before the OSCE, and quite a lot of the consultants working there were good, take you out of their way to have a look at this patient, now examine them, tell me what’s going on, that kind of thing. So quizzing you … Some of the consultants at [hospital] actually had a previous kind of RACE OSCE questions like on version PowerPoints. So call me in and like quiz me. So that was really going out of their way. (Trainee 14)*

Fifth, Directors of Training provided support in the form of information sessions for trainees on how to prepare for the examination, and monitored their progress throughout the preparation period. However, some trainees did not find the generic information helpful and would have preferred individually tailored advice delivered on a one-to-one basis with their Director of Training.

*I actually spent a lot of time with the final year registrars helping them prepare with their exams … lots of RACE tutorial trainings, so I’ve been involved in a lot of the teaching with the registrars, so setting up OSCEs, their conferences, their tutorials, and their sort of final exam preparation. (Supervisor/Director of Training 9)*

The College (RANZCO) was a sixth source of support, providing educational resources, curriculum standards, assigning mentors and facilitating access to psychological support. However, some trainees and supervisors commented that the College could provide more detailed guidance on how to prepare effectively for RACE.

*I do think that the College has great resources and has done a lot of supporting things for people and everyone has a mentor and things like that, so I do think that the College has done a lot for people. And also, I mean, from the sort of mental health point of view, there also is a lot of support. (Supervisor 3)*

*Maybe they need to do something about learning the exam technique, how do you answer this question safely and what are the red flags? And it doesn’t just have to be for those who fail, it could be something that everyone could learn before sitting. They don’t give lectures. They don’t give content. I find that a bit unusual. They just keep the curricular standards. (Supervisor 1)*

Lastly, and perhaps most importantly for trainees’ emotional and physical wellbeing, was the support provided by friends and family, and domestic service providers such as nannies and cleaners. Trainees reported that the support from friends and family was essential to maintain emotional wellbeing and study motivation. Trainees stated that they outsourced help when needed (e.g. home delivered meals, cleaning, childcare) to take the pressure off their home environment and maximise study time.

*I think first of all, I had incredible support. I think without [my partner’s] support, I would have really floundered. It was incredibly taxing to have the [children] around me wanting to be with me and for me not being able to be there. (Trainee 6)*

*My [partner] took on a lot, my mother took on a lot, everyone around me, we had a nanny, we had all hands onboard. We threw a lot of money at it. It was really hard but equally I think if I hadn’t put 100% in, I might not have got through and then I’d be dragging it out for another six months and then maybe even longer, so that was kind of my approach. (Trainee 18)*

### Get to know your opponent

Trainees largely described their familiarity with the format of the clinical component of RACE as they had the opportunity to attend mock clinical examinations prior to sitting. Given these are essentially simulated examinations, trainees felt well prepared to sit the clinical component of RACE.

*The clinical, I actually found it quite easy. In [network] we did a lot of OSCE kind of training all the time and really challenging cases as well and on the day, pretty much almost all the cases we had for the real exam were ones that we’ve already faced in a practice OSCE or in a teaching session. So we all found it pretty easy to be honest. (Trainee 14)*

However, the written component of RACE was described by interviewees as being unlike any university assessment they had previously encountered. Supervisors emphasised that the written component was not an examination of knowledge, but rather an assessment of how knowledge is applied. Some trainees only recognised this with hindsight, having failed RACE initially after expecting a traditional examination and hence preparing by focusing on reading textbooks to become saturated with knowledge. After reflecting on their failure, they described that they had not recognised the need to become familiar with the examination construct, and to develop and practise a specific examination technique to achieve success.

*I think one of the key things about the written paper is it’s an opportunity for the candidate to be able to write down their clinical approach and their clinical thinking and clinical reasoning, and that’s quite a complex thing for trainees to do because it’s really trying to put down what is going through their head when they’re seeing patients. (Supervisor 6)*

*I think just the big thing for me was doing lots and lots of old exam questions, going through them and then asking colleagues to look through them, or Fellows that had sat previously. I think that that was a big part of my preparation. (Trainee 9)*

All interviewees acknowledged the important role of using past examination papers as a study resource to prepare and develop examination specific technique. Practising past examinations allows trainees to become familiar with the examination format and understand the requirements to pass. In fact, some trainees specifically indicated that this was the sole mechanism used to prepare for the examination.

*What I did was I printed out every single old exam and the examiners’ feedback, and then I sat down and said, ‘what do the examiners want for each type of question?’ And then all I did was past exams for that entire period and if I didn’t know any information, I’d go and look it up. But I sat for every day and I did five written questions, 12 min each, that made 60 min and that was my 60 min of study per day. (Trainee 4)*

Written examination technique was reportedly best developed by using past examination papers to study, supported by robust discussion in study groups and obtaining feedback from Fellows, supervisors and examiners on written answer attempts. This helped trainees hone their technique by allowing them to identify patterns and templates for how to answer questions.

*I think just the big thing for me was doing lots and lots of old exam questions, going through them and then asking colleagues to look through them, either consultants or Fellows that had sat previously. I think that that was a big part of my preparation. (Trainee 9)*

*I worked out that the people passing quite efficiently were the people who just studied the exams … they structured their answers based on what the examiners wanted. (Trainee 11)*

Finally, trainees unanimously described the written component as being immensely time pressured. Therefore, they emphasised the importance of simulating the time constraints of examination conditions using past papers to develop efficiency in answering questions. Given the online nature of the written examinations, proficient touch typing was seen as essential.

*The first thing I did was actually a typing course … I spent a few months learning how to touch type in the evenings, just like 15 min … and I think that made a big difference, because there’s a huge time pressure and if you’re not a touch typist, I don’t know how you would do it. (Trainee 2)*

### There is no substitute for hard work

Trainees described that on average, RACE required at least twelve months of study to effectively prepare. Those trainees with young children reported that studying for longer periods was beneficial to counter the impact of family responsibilities on study time. Amongst those who sat RACE during the past five years, the shortest duration of study was eight months and the lengthiest was two years.

*About two years before [started studying for RACE], but not as intense for the first year. I was just taking my time going through things slowly and then more intense in the last 12 months. The main reason I did that is because I have children … I knew that I needed to get studying earlier as I’m not going to have as much time as a lot of my other colleagues. (Trainee 14)*

In terms of study intensity, trainees described dedicating at least one to three hours per day to RACE study individually, with additional group study sessions, tutorials and courses scheduled during the week. Trainees unanimously reported an increasing frequency and intensity of study leading up to the examination. Given the challenge of securing study time against work demands, some trainees reported being opportunistic in clinics, studying between patients and asking trainees in their basic years of training to shoulder more of the load to allow them opportunities to focus on studying.

*It’s pretty hardcore, to be honest, but like in terms of quantification, I would say it was basically in the lead up to the first one, it was three hours a day for a year. And that meant I used to get up at quarter past five and just be studying before work, trying to get in an hour or so before work, and then doing an hour or so or two after work. It’s just fairly constant. (Trainee 1)*

Most trainees described the need for a period of focused examination preparation in the immediate weeks before sitting, which was facilitated by either study or recreational leave. Some trainees consequently outlined their concerns that some training networks denied access to study leave immediately prior to the examination.

*I think it would be nice for all the sitting candidates to have that little bit of time off before, like two weeks or something where it’s just protected time off for those people sitting. Because it’s really stressful to think that you have to go into work the day before such a big [examination]. To allow the candidates to really focus on that, I think it would be nice to have leave before the exam. (Trainee 18)*

Noting the duration and intensity of preparation, interviewees almost universally described the RACE preparation period as being the single most physically, mentally and emotionally challenging time of their educational journey. Trainees therefore almost unanimously reflected on their ‘one and done’ attitude, whereby they wanted to give themselves the best chance of passing first sit by throwing everything they had at the initial preparation process.

*It was quite stressful and quite intense, but I managed to get through and my experience, I guess I had a lot to lose, so I threw everything at it. I just didn’t want to have to do it again. (Trainee 18)*

Interviewees emphasised that self-determination underpinned preparation efforts that were necessary for success in RACE. Supervisors described some situations where they felt trainees were not as dedicated as they needed to be during the preparation period, and that more efforts were needed to educate trainees on the importance of being proactive and self-directed in their preparation.

*You really have to drive it yourself I think is the thing. You have to not expect anybody’s going to give you anything … I think if you go in with the attitude that I’ve got to really do all this myself, then I think that gives you the best chance of passing. (Trainee 16)*

*You’ve got to do everything you can. You’ve got to ask everyone you come across, for a tutorial, for some help, for some exam question marking, or just talking through scenarios. You’ve got to be proactive and maybe the trainees don’t hear that from enough people? (Supervisor 8)*

Mechanisms that trainees reported as helpful for managing the ongoing stress and pressure included daily exercise where possible, eating well, outsourcing help when needed, and using mindfulness techniques. Recognising the need to take a break from study to support mental and physical health was also emphasised during the preparation period, but often this proved counterproductive.

*You take a bit of a break because you know you need a break, but every time while you’re taking that break, you’re always feeling guilty thinking ‘I should be studying’. (Trainee 19)*

### Keep pace with the herd

Benchmarking efforts against other trainees preparing to sit was important in the preparation process, which related to trainees reflecting on both the amount of time spent studying, and their knowledge base and understanding of examination technique relative to their peers. Group study was an important conduit for benchmarking, as it highlighted expectations for study effort and also offered the opportunity to share knowledge, study timetables and materials. Trainees described the inherent downplaying of time spent studying by their peers, which meant that some sitting candidates genuinely didn’t appreciate how much effort people were putting into preparation.

*I have a friend who’s failed a few times and I think one of the things, especially the first time they sat it, they just had no idea how much people studied for it, or like how long they studied for it and things like that. Like I said, everybody plays down how much they study. (Trainee 2)*

Benchmarking was also achieved through trainees attending specific ophthalmology training courses and conferences during RACE preparation. The Dunedin Ophthalmology Clinical Course in particular was almost unanimously viewed as critical for RACE preparation. However, it is important to note that not all interviewees attended Dunedin, with one describing the belief that attendance was not necessary for success. Trainees who attended the course described the opportunity to develop and refine their examination technique, identify gaps in their learning and benchmark their level of knowledge against others preparing to sit RACE.

*I think Dunedin [course] is invaluable. I mean, you’re living, breathing, eating, sleeping ophthalmology and when you finish, you just leave thinking, ‘what did I even know before? … It’s just great to consolidate all of that knowledge and information, and have like-minded people pushing you to your limits. (Trainee 3)*

### Don’t jump the gun

Finally, interviewees highlighted the importance of being adequately prepared to meet examination standards, rather than attempting RACE as soon as eligible. The temptation to sit at the first available opportunity often reflected the immense pressure and intensity that accompanied the preparation period, and the desire of trainees to reclaim their personal life. Trainees described becoming eligible typically at the end of their third year of training. However, interruptions to training due to health or family reasons, as well as the timing of opportunities to access certain training rotations, meant that some trainees had not had sufficient clinical experience or time to prepare. One trainee recognised their hesitation at sitting so close to their initial eligibility, but described being encouraged by others given the notion that there were multiple opportunities to pass.

*I was a few weeks shy of the three-year mark when I sat, but I felt ready enough … I had plenty of time to study and I guess people said to me ‘look, two schools of thought – you sit early and often, or you sit once and done’. I was quite nervous about it, but then so many people encouraged me just to get on and give it a go that I was like, ‘okay, if you all think it’s a good idea, I’ll give it a go.’ And so I was able to pass the clinical at that point, but I didn’t pass the written … It was quite demoralising, obviously, failing the first round. (Trainee 13)*

Supervisors shared that they could often predict which trainees were not ready based on clinical performance and preparation, and consequently encouraged these trainees to consider delaying their RACE attempt. Interviewees conceded that a face-to-face meeting prior to the intention to initially sit RACE was therefore a critical step in promoting success as it allowed the opportunity to assess the trainee’s preparation progress and readiness to sit. However, supervisors described that advice given to delay sitting due to concerns about preparation was largely ignored, which in some cases resulted in examination failure. Supervisors emphasised the importance of trainees heeding their advice and waiting until they were properly prepared to maximise chances of success.

*The Director of Training said up front, ‘don’t do the exam in the next sitting, you’re not ready for it, you are going to fail it.’ And so [they] deferred [the] exam and passed both exams first go … Incidentally, one other person in [training network] that year also got told ‘you’re not ready to sit the exam.’ But that person sat anyway and failed … I think it’s worthwhile if RANZCO suggests to all Directors of Training that every time you have trainees approaching you saying, ‘I want to sit RACE’, you sit down with them for about 10 min and actually make sure they’re ready. (Trainee 3)*

## Discussion

We identified six key approaches to preparation that promoted ophthalmology trainee success in their final summative examinations: developing and adhering to a clear study plan; trainees establishing and activating personal and professional supports; developing an understanding of the examination construct, format and requirements; diligence in committing to an extended period of intensive study; benchmarking preparation efforts and progress against peers to ensure parity of effort and knowledge; and ensuring readiness to sit. These findings are pertinent for trainees approaching their final summative examination, regardless of medical speciality. The findings are also relevant for supervisors, training networks, specialist Colleges and health systems, by describing the strategies which support success on final summative examinations.

The approach, encapsulated by the quote ‘Those who fail to plan, plan to fail’, related to the importance of trainees adequately planning their study schedule to ensure that they had sufficient time and opportunity to cover the curriculum standards and consolidate their clinical knowledge. This is supported by Evans and Suetini, who noted that the adage ‘Plan the work and work the plan’ is crucial for the success of psychiatry trainees sitting their final summative examinations [24]. Other research has also reported that timetable-based study is associated with examination success [16, 25]. Participants described developing and rigorously adhering to study plans, both individually and within groups. However, it was noted that training posts in certain locations had excessive clinical workloads which were an unavoidable diversion away from planned study. Savvy trainees sought information about the clinical workloads of various training locations and planned their placements to maximise study time during the period leading up their final examinations. These efforts may be actively supported by training networks and health systems, through ensuring trainees preparing to sit RACE are allocated to training rotations that offer flexibility to study and limited on-call obligations.

We found that ‘It takes a village’ to prepare and support specialist medical trainees for their final summative examinations. Several studies have described peer study groups as pivotal to examination success [7, 21, 25, 26]. Beyond the immediate benefits for examination preparation, study groups provide psycho-social support during a stressful period of the trainee’s career. Our findings add to the literature by highlighting the importance of the broader collective training network, the College and trainees’ social support networks in supporting trainees throughout the preparation period. Specialist training Colleges and training networks could ensure that appropriate policies and processes are in place to foster a professionally supportive environment for trainees in their advanced years of training. This includes arranging study groups if necessary, especially online study groups for isolated trainees in rural rotations, directing trainees in their basic years of training to deliver routine clinics to maximise in-hours study time for sitting trainees, working with training networks to ensure that trainees have protected study time to attend tutorials and lectures, and assigning every trainee a mentor who has recent experience on the College examination board. Reciprocity was especially important for facilitating study efforts, and hence specialist Colleges should actively seek opportunities for all trainees, regardless of training year, to get to know one another to build the support network necessary for success. All supervisors and Fellows in training networks, together with Directors of Training have key roles in supporting trainees through offering advice on written examination efforts and quizzing them on patients in clinics. Colleges must also play a role in facilitating examination preparation by ensuring trainees have access to information regarding the examination and resources to help guide preparation. Finally, a strong network and family and friends are necessary to provide social supports for the lengthy and stressful preparation period.

‘Get to know your opponent’ encompassed trainee understanding of the examination construct, format and requirements, which was universally understood amongst interviewees in our study as critical to examination success. Practising past examination questions, supported by robust discussion in study groups and approaching consultants for feedback, was reported to be a key component of attaining an understanding of the examination format and the correct technique of applying clinical knowledge in answering examination questions. Indeed, the use of previous examinations as a study tool is common among trainees preparing for specialist examinations [7, 24–26]. Evans and Suetani describe this strategy as the ‘backbone’ of examination preparation [24]. Khanna et al. reported that access to past examination questions and model answers is often dependent upon collegial relationships with previous candidates [19]. Specialist training Colleges can ensure fairness by making past examination questions and model answers available to all trainees. The recent adaptation to online examination also presents new challenges for trainees, especially those lacking typing proficiency. Simulating examination conditions with time pressure and typing requirements will auger well for trainee preparation.

Interviewees unanimously perceived that ‘There is no substitute for hard work’ when it comes to examination preparation. This effort is largely the result of self-determination and commitment. Trainees need to ensure a balance between theoretical examination preparation and clinical skills development. Theoretical study should not be prioritised at the expense of clinical skills development. Trainees and supervisors are jointly responsible for ensuring clinical skills requirements are met during each rotation.

Specialist trainees described difficulties balancing clinical workloads, study requirements, personal responsibilities and family obligations during the prolonged and all-consuming process of preparing for specialist final examinations. This research and other studies have found that personal relationships often become strained and burnout is common [6, 7, 14]. Trainees need to implement strategies to foster wellbeing during this period of intense stress. This may include a period of annual leave before they commence their examination preparation to ensure they begin the process in optimal physical and mental health. Training networks should ensure equitable access to study leave immediately prior to final examinations, so that trainees can focus on study. This is important given the high-stakes nature of final assessments.

We identified that ‘Keeping pace with the herd’ was an important strategy during the preparation period, whereby trainees needed to benchmark their study efforts, knowledge and progress against their peers. Trainees made the point that not everyone is truthful about the effort being put into study, meaning that those studying by themselves may be genuinely unaware of the commitment and dedication required to achieve. Benchmarking was often achieved through group study environments where trainees could see firsthand the effort being put in by fellow candidates, and could reflect on their own mock examination answers relative to others. The Dunedin Ophthalmology Clinical Course was also seen as another important vehicle for benchmarking, especially to identify personal gaps in content and understanding.

Similar to previous research [26, 27], readiness to sit was a critical factor for success in RACE. Ensuring trainees ‘Don’t jump the gun’ is the responsibility of trainees themselves, supervisors and training networks. Readiness to sit should ideally be assessed during a formal meeting between the trainee, their supervisory team and the Director of Training when completing intention to sit documentation to ensure that the trainee has the skills and knowledge to pass the examination, and that they are psycho-socially prepared to embark upon the exhausting study regimen required for examination success. The advice of supervisors and Directors of Training must be taken into account to avoid prematurely attempting the examination when the candidate is not adequately prepared. Trainees need to reflect on their training progress, knowledge of the curriculum, clinical skills and personal circumstances to determine whether they are ‘ready to sit’. Ultimately, delaying an examination attempt to further prepare may end up being the lesser of two evils when considering the potential impact of examination failure.

## Study limitations

As our study examined the experiences and perspectives of participants who self-selected to be interviewed, the findings cannot claim to be representative of all ophthalmologists who have sat RACE in the past five years or supervisors who have supported them to prepare and succeed across Australia and New Zealand. However, the analysis showed a broad range of perspectives and the results were not dominated by a small group. Further, it may be that those participants who had a negative experience of the final summative examination were more motivated to participate. Finally, those who failed at their first attempt may have engaged in deeper reflection about the reasons for their initial failure, and later success, compared with those who passed at first attempt. However, some participants who passed at first attempt also reflected deeply on the examination and were interested to know which questions they failed so that they could improve their knowledge in these areas to ensure safe clinical practice. Including all eligible trainees, regardless of failure, was important to capturing the breadth and depth of experiences.

## Conclusions

It is in the best interest of trainees, training Colleges, health care systems and communities to maximise passing rates on final summative examinations in medical specialist training, whilst maintaining clinical and professional standards. Our study identified six approaches to preparation which can support trainees achieve success on RACE. Training Colleges can facilitate these preparation approaches by formalising readiness to sit appraisals, and providing equitable access to past examination questions, model answers and expert feedback from consultants on examination boards. Training networks and health systems can assist preparation for trainees by implementing strategic training pathways, where trainees are allocated rotations with manageable clinical workloads and access to study leave during the months leading up to their final summative examination. Fostering collegiality within networks from the inception of training will also bode well for examination success given the extra support trainees need when preparing to sit. Finally, trainees can adhere to successful preparation approaches by adequately preparing for final summative examination, measuring their efforts against others, and demonstrating self-determination to achieve the level of success required in RACE. Collectively, these efforts from all stakeholders will improve the medical specialist training pipeline and help to address medical specialist workforce shortages.

### Electronic supplementary material

Below is the link to the electronic supplementary material.

Supplementary Material 1

## Data Availability

The dataset obtained and analysed during the current study is available from the corresponding author on reasonable request.

## List of abbreviations

OSCE: Objective Structured Clinical Examination
RACE: Royal Australian and New Zealand College of Ophthalmologists Advanced Clinical Examination
RANZCO: Royal Australian and New Zealand College of Ophthalmologists
