# Supplementary material for: Preparing for success in final summative medical specialist examinations: The case for RACE
Source: BMC Med Educ. 2023 Dec 5;23:918. doi: 10.1186/s12909-023-04920-y (PMC10696859; doi:10.1186/s12909-023-04920-y)
Supplement: Supplementary file 1 — Supplementary Material 1 [file 12909_2023_4920_MOESM1_ESM.docx]

**Promoting Success in the RANZCO Advanced Clinical Examinations (RACE)**

**Pre-amble**: Introduction, thank you for agreeing to participate, purpose of the study, verbal consent, audio record interview, invitation to check transcript and add/revise statements

**Opening Question:**

Could we start by you telling me little about your experience of the RACE exams – both the clinical and the written components?

**Prompts for trainees:**

- Do you feel that you did better on one exam (e.g. clinical) than the other? Why was this?
- How closely do you feel the exam questions and stations reflected your training experiences at the time? Were you able to draw on your clinical experience as a trainee to help answer questions?
- How did you prepare for these exams? Overall, what impact did this have on your wellbeing and ‘work-life’ balance?
- Do think there are aspects of the RACE that could be improved? What might these be? (e.g. format, duration, timing, balance between very short, short and long answer questions, level of difficulty, relationship to clinical practice, understanding the questions/expectation of the examiner)
- Did your Director of Training or supervisors give any advice on how to answer the questions in the written paper? What did they suggest?
- What should be done (including by RANZCO) to better support trainees preparing for RACE?

**Prompts for supervisors:**

- What do you think are the reasons for some trainees struggling to pass RACE?
- Have you noticed any differences between trainees who pass RACE and those who fail?
- Would you be able to identify trainees as being more likely to fail before they sit the exam and, if so, what do you think are the more common issues?
- How are trainees being supported by their trainee peers, post supervisors and training networks to help them prepare for RACE?
- How can trainees preparing for RACE be supported better?
- What should RANZCO do at a federal level to support trainees to better prepare for RACE?
